# Orthodontic Treatment Need and the Psychosocial Impact of Malocclusion in 12-Year-Old Hong Kong Children

**DOI:** 10.1155/2019/2685437

**Published:** 2019-06-12

**Authors:** Derek Baram, Yanqi Yang, Chong Ren, Ziling Wang, Ricky Wing Kit Wong, Urban Hägg, Colman McGrath, Min Gu

**Affiliations:** ^1^Department of Orthodontics, Faculty of Dentistry, The University of Hong Kong, Hong Kong; ^2^Faculty of Dentistry, The University of Hong Kong, Hong Kong; ^3^Department of Dentistry and Maxillofacial Surgery Cleft Center (Craniofacial Orthodontics), United Christian Hospital, Hong Kong; ^4^Department of Public Health, Faculty of Dentistry, The University of Hong Kong, Hong Kong

## Abstract

**Objectives:**

To determine the prevalence of orthodontic treatment need in 12-year-old children in Hong Kong and its relationship with the psychosocial impact of malocclusion and to assess their associations with sociodemographic factors.

**Materials and Methods:**

A random sample of 687 12-year-old children was recruited from 45 secondary schools in Hong Kong. Orthodontic treatment need was assessed on study models by five indices: the Dental Health Component of the Index of Orthodontic Treatment Need (IOTN-DHC), the Aesthetic Component of the IOTN (IOTN-AC), the Dental Aesthetic Index (DAI), the Index of Complexity Outcome and Need (ICON), and the Peer Assessment Rating (PAR). The psychosocial impact of malocclusion on participants and sociodemographic factors were obtained from a questionnaire. Logistic regression was used to examine the correlations between treatment need and the psychosocial impact of malocclusion as well as their associations with sociodemographic factors.

**Results:**

The final number of participants was 667 (339 boys and 328 girls, participation rate 667/687 = 97.1%). The prevalence of orthodontic treatment need varied depending on the indices used (10.9–47.8%), but significant correlations were found among the five indices (*p* < 0.01). The uptake of treatment among the cohort was 2.3%. Boys had higher IOTN-DHC (*p* < 0.05), DAI (*p* < 0.05), and PAR (*p* = 0.05) scores than girls. IOTN-AC was significantly associated with the psychosocial impact of malocclusion (*p* < 0.05). Parents' level of education and household income were not significantly associated with either treatment need or the psychosocial impact of malocclusion (*p* > 0.05).

**Conclusion:**

The need for orthodontic treatment in 12-year-old children in Hong Kong remained high, and the uptake of treatment was low. Boys had a higher normative treatment need than girls. Among the five indices, IOTN-AC appears to be the best indicator of the psychosocial impact of malocclusion.

## 1. Introduction

The assessment of orthodontic treatment need is often necessary in epidemiological studies in order to assist with the allocation of public health resources to those who are most in need [1–5]. In private dental practices, it could also help orthodontists to assess the severity of malocclusions and to evaluate treatment outcomes [2, 5]. Various orthodontic indices have been developed for such assessment over the past several decades [6–9]. Today, the most commonly used orthodontic indices include the Index of Orthodontic Treatment Need (IOTN), the Dental Aesthetic Index (DAI), the Peer Assessment Rating (PAR), and the Index of Complexity Outcome and Need (ICON) [10].

The IOTN is based on modified versions of the priority Index of Orthodontic Treatment Need used by the Swedish Dental Health Board [11] and the SCAN Index [12]. It comprises two parts: the Dental Health Component (DHC), with five severity levels, and the aesthetic component (AC), with ten severity levels [7]. The DAI sums up the scores of clinical and esthetic components to obtain a single value, which can then be categorized into one of the four severity levels to give an indication of treatment need [4, 6]. The PAR summary score is a weighted combination of seven occlusal traits. The treatment outcome can be assessed by the percentage change comparing pre- and posttreatment scores [8]. The ICON is a multipurpose index for assessment of orthodontic treatment need, complexity, improvement, and outcome using a single set of occlusal traits and is designed to be a more comprehensive, user-friendly, and internationalized alternative to the IOTN and PAR [9].

These indices differ in multiple aspects, including the choice of traits for assessment, the weighting factors for each trait, and the cut-off values for severity grading [1, 4, 6–9]. Therefore, the prevalence of orthodontic treatment need can vary considerably when different indices are used for assessment [13–15]. However, the indices do share some common traits for identifying treatment need; thus, there have also been reports of significant correlations between some indices [13, 14, 16, 17].

In some countries, including the United Kingdom, the need for and the uptake rate of orthodontic treatment among children are assessed regularly by the government in order to understand the prevalence and severity of malocclusion as well as its possible burden on the family and society [18]. In Hong Kong, however, the current oral health survey conducted by the government has not included the assessment of orthodontic treatment need [19]. Therefore, similar information can only be estimated from limited previous studies [20–22].

A 1999 survey of 765 12-year-old Hong Kong children showed that 37% have a definite treatment need (IOTN-DHC: categories 4 and 5) [22]. The uptake rate, however, was low when compared with high treatment need, as only 1% of these children had received or were undergoing orthodontic treatment [22]. Some more recent research indicated that over one-third of young adults in Hong Kong needed orthodontic treatment [20, 21]. In 2001, a study of 223 young Chinese adults aged between 18 and 24 found that 54% had “great” or “very great” orthodontic treatment need according to IOTN-DHC [20]. In 2009, another study of 120 young Hong Kong adults reported the prevalence to be 33% [21].

The prevalence of psychosocial demand for orthodontic treatment is often different from that of the normative treatment need [14, 17, 23–27]. While the orthodontic indices are mostly designed from the clinician's perspective, the possible psychological impact of malocclusion is a subjective feeling of the patient. The previously reported orthodontic treatment need in Hong Kong was often different from patients' demand for treatment [14, 20–22, 27]. In the 1999 survey targeting 12-year-old Hong Kong children, two-thirds of participants were not satisfied with their dental appearance, which is much higher than the 37% normative treatment need found in the same study [22].

This study aimed to provide more updated information about the prevalence of orthodontic treatment need among 12-year-old Hong Kong children. Correlations among the five proposed orthodontic indices (IOTN-DHC, IOTN-AC, DAI, PAR, and ICON), whether they are able to indicate the psychosocial impact of malocclusion, and their associations with sociodemographic factors are also investigated and analyzed in this study.

## 2. Materials and Methods

### 2.1. Sample Selection

The sample of this cross-sectional study was part of a Chinese birth cohort in Hong Kong named “Children of 1997”. The cohort comprises 8,327 ethnic Chinese infants born between April and May 1997, corresponding to about 88% of all infants born during that period [28]. This is an excellent representative randomized sample of children growing up after the handover of Hong Kong, which has posed significant changes in the education, culture, economics, and many other aspects of the city. Numerous data from these children, including their medical and dental records, were collected at the time of their birth, and the children are still continuously monitored for different research [28]. The cohort remains an important representative sample for Hong Kong. Advantages of using this cohort include the close proximity of the participants' age and the fact that they could be easily tracked down in future for any further research on malocclusion. Ethics approval was obtained from the Institutional Review Board of the University of Hong Kong/Hospital Authority Hong Kong West Cluster (UW 09-453).

Forty-five secondary schools (accounting for about 10% of all local secondary schools in Hong Kong) from 18 districts of Hong Kong were randomly selected using stratified random sampling method. Schools were stratified by districts, and within each stratified unit, random sample of schools was chosen using simple random digits. All 687 Form 1 and Form 2 students (corresponding to Grades 7 and 8 in the North American-based system) in these selected secondary schools born between 1 April and 31 May 1997 were invited to participate in the study. Considering a 95% confidence interval, previously reported prevalence of orthodontic treatment need (33–54%) [20–22], and a potential nonparticipation rate of 25%, the minimum sample size was set at around 510 participants.

From January to May 2010, a group of dentists obtained study models and completed questionnaires from the participants for further assessment. As an incentive for participating in the research project, a comprehensive dental and orthodontic report was later sent to the parents.

### 2.2. Assessment of Orthodontic Treatment Need

To assess the normative treatment need, the five indices were used to analyze all of the models, following the standard cut-off values suggested by the original authors: IOTN-AC: categories 8-10 [1, 7], IOTN-DHC: categories 4 and 5 [1, 7], DAI: scores 36 and above [1, 4], and ICON: scores greater than 42 [9]. The cut-off value for the PAR was based on the PAR nomogram: for a malocclusion to have the potential of being “greatly improved” it must have an initial score of 25 or above [8].

### 2.3. Psychosocial Impact of Malocclusion and Sociodemographic Information

A questionnaire was designed to elicit the psychosocial impact of malocclusion and sociodemographic information.

The psychosocial impact of malocclusion on a participant was assessed by answers to three questions:

(1) “In the past 3 months, have you been concerned with what other people think about your teeth, lips, mouth or jaws?”

(2) “In the past 3 months, have you avoided smiling or laughing when around other children because of your teeth/mouth?”

(3) “In the past 3 months, have you been teased or called names by other children because of your teeth/mouth?”

The three questions were extracted from of short versions of Child Perception Questionnaire for 11-14-year-old Children (CPQ_11-14_) [29]. The first question was from emotional well-being domain and the other two questions were from social well-being domain. All of these three items could reflect the social judgments.

Three sociodemographic factors were collected and analyzed in this study: sex, parents' level of education, and monthly household income. The cut-off value for monthly household income was set at 20,000 Hong Kong dollars (HKD) (≈2,500 USD). Parental education level was divided into two groups: those with a college education or above and those with an education below college level.

### 2.4. Statistical Analysis

Logistic regression analysis was used to examine the correlations (1) among orthodontic treatment need measured by the five indices, (2) between treatment need and the psychosocial impact of malocclusion, (3) between orthodontic treatment need and sociodemographic factors, and (4) between the psychosocial impact of malocclusion and sociodemographic factors.

### 2.5. Method Error

Two calibrated examiners analyzed all the study models. Interexaminer reliability for the five indices was determined by assessing 50 models for calibration and then comparing the results with the assessment of a further 50 models to calculate the unweighted kappa scores, because orthodontic treatment needs are binary data (need/no need). Intraexaminer reliability was analyzed by repeating assessment of 10 models after each set of 100 models and comparing the results. The kappa scores to assess interexaminer reliability were 0.60, 0.67, 0.67, 0.47, and 0.56 for the IOTN-AC, IOTN-DHC, DAI, ICON, and PAR, respectively.

## 3. Results

Six hundred and eighty-seven adolescent participants were recruited for the primary sample, and only one individual later refused to participate in the research. Out of the 686 participants in the secondary sample, 16 (2.3%) had received or were undergoing orthodontic treatment and were excluded from the study model analysis. Fractured incisors were found in a further three models. Therefore, 667 adolescent participants (339 boys, 328 girls) were finally recruited for assessment. The participation rate was 97.1%.

The definite need for treatment as assessed by the five indices varied from 10.9 to 47.8% (Table 1). Although the range of treatment need varied considerably among the indices, the correlations between the indices were found to be moderate (*p *< 0.01) (Table 2). For the psychosocial impact of malocclusion, 60% of the children were concerned about what other people thought of their teeth, 34% had avoided smiling and laughing, and 48% had been teased or called names because of their teeth (Table 3). Among the five indices, only IOTN-AC showed a significant association with the psychosocial impact of malocclusion (*p* = 0.04, 0.02, 0.09, respectively) (Table 3).

Three indices, the IOTN-DHC, DAI, and PAR, indicated that boys tended to have more severe malocclusion than girls (*p *= 0.02, 0.03, 0.05, respectively). Among all the participants, 79% of their parents had an education level below college, and 57% of households had an income below 20,000 HKD per month. Only two indices, the IOTN-DHC and PAR, showed a statistically significant association with the parents' level of education (*p *= 0.02 for both indices). None of the indices showed a significant association between normative treatment need and monthly household income (Table 4). No statistically significant association was found between sociodemographic factors and the psychosocial impact of malocclusion (Table 5).

## 4. Discussion 

### 4.1. Orthodontic Treatment Need in Hong Kong

This study confirmed that there is a high need for orthodontic treatment in Hong Kong, which is generally in agreement with the findings of previous Hong Kong studies [20–22]. The distribution of the IOTN-DHC scores in this study was similar to that observed in 12-year-old children 20 years ago [22]. As each index measures treatment need differently, the wide range of need (10.9–47.8%) is unsurprising (Table 1).

Nevertheless, there was a significant moderate to strong correlation between the indices, suggesting that these occlusal indices do share some common traits in assessing orthodontic treatment need (Table 2). In particular, there was a strong correlation (*r *> 0.7) between IOTN-AC and ICON scores, because the IOTN-AC is incorporated in and has a heavy weighting in the ICON analysis [9]. Similar correlations between IOTN-AC and ICON were also found in previous studies. Two studies in Hong Kong investigating correlations between IOTN-AC, IOTN-DHC, ICON, and DAI also found that the strongest correlations were between IOTN-AC and ICON (*r *> 0.8 in both studies) [13, 14].

The weakest correlation was found between IOTN-DHC and DAI (*r* = 0.495). It is compatible with a study in Hong Kong by Zhang et al., where the weakest correlation was also between IOTN-DHC and DAI (*r *= 0.32) [14]. Such weak correlation could be explained by the nature of DAI, as it lacks assessment of some occlusal traits including crossbite, deep overbite, and centerline discrepancy [30]. Moderate correlation was found between IOTN-DHC and IOTN-AC (*r *= 0.544), which is compatible with previous studies (*r *= 0.37-0.60) [13, 14, 17, 31]. It is not surprising to find some discrepancies between DHC and AC of IOTN, as some dental health implications do not necessarily result in esthetic impairment and high AC grades [30]. Also, IOTN-AC is based on subjective evaluation of esthetic only. Although examiners in present study have been calibrated, examiners in different studies could have variable subjective perceptions, for example, due to cultural differences [32].

Correlations between some other indices were in disagreement with previous studies. Only a moderate correlation was found between DAI and IOTN-AC (*r* = 0.567), although both indices were heavily based on aesthetic component. This is in disagreement with a previous study of 728 Iranian teenagers, where a strong correlation was found between two indices (*r* = 0.795) [33]. IOTN-DHC showed a moderate correlation with ICON in current study, while other studies have shown strong agreement between the two indices [31, 34]. However, since these studies did not include other orthodontic treatment need indices, no comparisons were possible between different correlations and variations in different study samples. Also, ethnic differences might partially explain such disagreement.

The inconsistency of these results highlights the limitations and differences of these indices. Although the literature suggests general agreement among the indices, many differences remain. It would be prudent for the clinician or service provider to assess the results of these indices with a degree of caution and to recognize that they may not fully reflect the psychosocial or clinical effects of the malocclusion.

### 4.2. Uptake of Orthodontic Treatment in Hong Kong

Although the need for orthodontic treatment in Hong Kong has remained high over the years, the uptake of such treatment is low. In 2002, an assessment of university students in Hong Kong found that only 3.9% had received orthodontic treatment [20]. In 2009, another study of 240 university students in Hong Kong revealed an uptake rate of 13% [21]. This is comparatively lower than the uptake rate of about one-third reported in similar age groups in Western societies [35, 36].

For 12-year-old children in Hong Kong, the uptake rate in 1999 was shown to be only 1% [22], while in the same year, 12% of 14-year-old children in the United Kingdom were undergoing or had received orthodontic treatment [24]. A more recent study in Spain showed an uptake rate of 23.5% among 12-year-old children in 2010 [37]. Another government report in the United Kingdom also indicated that 9% of 12-year-old children were undergoing orthodontic treatment in 2013 [18]. While targeting the same age group, both results were much higher than the uptake rate found in this study (2.3%).

The low uptake rate for orthodontic treatment may be related to the mode of the dental service system in Hong Kong, especially in the field of orthodontics. Currently, there are no subsidized orthodontic services in Hong Kong except for civil servants and their dependents and for the limited number accepted by university dental clinics as teaching patients [38]. Others can only seek orthodontic treatment in private dental clinics. The cost for comprehensive orthodontic fixed appliances in Hong Kong is around 45,000–70,000 HKD (≈5,700-9,000 USD) according to the fee schedule for private patients in a public dental hospital [39], while Hong Kong's median individual monthly employment income was only around 15,000 HKD (≈1,900 USD) in 2016 [40]. As one of the cities with the greatest disparity of wealth in the world [41], Hong Kong in 2010 had 17.9% of its population living in poverty, with about one in four children living in low-income households [42]. Therefore, it is not surprising that private orthodontic services are usually reserved for the more privileged minority in spite of a high treatment need and demand among the population.

### 4.3. Association between Treatment Need and the Psychosocial Impact of Malocclusion

A lack of agreement between treatment need and the psychosocial impact of malocclusion has been well documented in the literature. In most cases, patients who have a high treatment demand present with a low normative need and vice versa [20, 22]. In this study, only the IOTN-AC showed evidence of a relationship with the psychosocial impact of malocclusion. Another study in the United Kingdom of 50 consecutively treated cases found that only the IOTN-AC predicted the psychosocial impact of malocclusion, while the IOTN-DHC and DAI had only weak associations [17]. Other investigations in Hong Kong have also found significant relationships between the IOTN-AC and the self-perception of malocclusion [13, 14]. The association between the two variables is not surprising, as the IOTN-AC was designed solely to assess the psychosocial effect of malocclusion on the individual [12]. Such a correlation has also been demonstrated in other previous studies [23, 43].

The other four indices appeared to be poor indicators of the psychosocial impact of malocclusion. Although the DAI and ICON are meant to take psychosocial factors into account [1, 9], they were not sufficiently sensitive in this study (Table 3). It is perhaps not surprising that the IOTN-DHC and the PAR failed to show an association, because the two indices are designed for clinicians to assess normative treatment need and treatment outcomes, respectively [7, 8].

### 4.4. Association between Treatment Need and Sociodemographic Status

#### 4.4.1. Sex

This study shows that boys tend to have a higher treatment need than girls at age 12. The reason for this phenomenon could be that on average, skeletal development in girls is roughly 2 years more advanced than in boys [44], resulting in boys having a greater proportion of Class II malocclusions at age 12 [45]. Some previous studies have also reported similar findings. In a study of more than 1,500 12-13-year-old Malaysian children, girls were found to have lower DAI scores than boys [46].

#### 4.4.2. Parents' Level of Education and Monthly Household Income

As a large proportion of malocclusion is dictated by genetic factors [47], it may at first seem improbable to assume that socioeconomic status and treatment need are related, yet there have been studies that suggest such an association. A significant association was found between malocclusion and the subjects' area of residence (urban/rural) in a study in Malaysia [46]. In a recent study carried out in Turkey, researchers compared parents' income and education level with children's normative need assessed by orthodontists using the IOTN-DHC, and the underprivileged were found to have higher IOTN-DHC scores [48]. Tickle et al. [24] stated that “undefined mechanisms” were responsible for the association between the prevalence of malocclusion and socioeconomic background.

Such associations between malocclusion and socioeconomic status as previously reported could perhaps be explained by a higher prevalence of caries in certain underprivileged populations [49]. A study in Hong Kong has also found a significant correlation between caries and socioeconomic background [50]. Caries could result in the early loss of the deciduous dentition and thus might ultimately increase the prevalence of malocclusion [51].

This study, however, did not find a strong link between treatment need and level of education or monthly household income. Although the IOTN-DHC and the PAR both showed a significant association with level of education, the other indices did not. Thus, the findings may have occurred by chance (Table 4). One possible explanation for this inconsistency with previous findings is that perhaps previous studies that have found such an association had assessed socioeconomic status without excluding potential confounders such as ethnicity differences, which had been demonstrated to affect orthodontic treatment need in several previous studies [26, 52, 53]. For example, they could have used participants' postcodes or areas of residence to classify certain districts into areas of wealth [46], and as certain ethnicities are more prevalent in particular districts, the trend could be a reflection of this factor rather than socioeconomic status.

The limitations of this study include the assessment of occlusion with the aid of study models only. The participants' facial appearance and dynamic occlusion were not considered. Therefore, in the IOTN-DHC analysis, participants with crossbite or reverse overjet were considered to have mandibular displacement or masticatory and speech difficulties, respectively, thus placing more participants in a higher treatment need category than may have been the case with a clinical assessment [7]. Furthermore, without the use of radiographs, hypodontia or impacted teeth may have been missed or wrongly assumed. Another limitation is related to the use of PAR, as it was first developed to measure treatment outcome rather than treatment need [8]. The assessment of treatment need with a cut-off value of 25 derived from its nomogram was only an indirect measurement. Use of a different cut-off value could therefore result in different findings. The assessment of the psychosocial impact of malocclusion is always complex and fraught with difficulties [54]. Participants may have felt that the appearance or color of their teeth and gums affected their self-confidence rather than their occlusion. Also, only three questions from CPQ_11-14_ were used to evaluate psychosocial impact of malocclusion. Using a more comprehensive version of the questionnaire could provide more accurate assessments. The participants from “Children of 1997” birth cohort were all ethnic Chinese. Although most of ethnic Chinese are Han Chinese, there are also many ethnic minorities. This might affect the homogeneity of participants' ethnic background.

## 5. Conclusion

(1) The orthodontic treatment need of 12-year-old children in Hong Kong remained high while uptake of treatment was low.

(2) Boys had a higher normative need than girls at the age of 12.

(3) Among the five indices, the IOTN-AC appears to be the best indicator of psychosocial impact of reasonable request.

## Figures and Tables

**Table 1:** Normative orthodontic treatment need assessed with the IOTN-AC, DAI, ICON, and PAR.

	No definite treatment need	Definite treatment need
IOTN-AC	89.1% (*n* = 594)	10.9% (*n* = 73)
IOTN-DHC	52.2% (*n = *348)	47.8% (*n* = 319)
DAI	69.4% (*n* = 463)	30.6% (*n* = 204)
ICON	62.1% (*n* = 414)	37.9% (*n* = 253)
PAR	88.6% (*n* = 591)	11.4% (*n* = 76)

Definite treatment need: IOTN-AC: categories 8-10, IOTN-DHC: categories 4 and 5, DAI: score **≥**36, ICON: >42, PAR: initial score **≥**25.

**Table 2:** Associations between the IOTN, DAI, ICON, and PAR.

	IOTN-DHC	DAI	ICON	PAR
IOTN-AC	0.544*∗∗*	0.567*∗∗*	0.722*∗∗*	0.584*∗∗*
IOTN-DHC	-	0.495*∗∗*	0.569*∗∗*	0.554*∗∗*
DAI	-	-	0.605*∗∗*	0.577*∗∗*
ICON	-	-	-	0.657*∗∗*
PAR	-	-	-	-

*∗∗p *< 0.01.

**Table 3:** Associations between normative treatment need and the psychosocial impact of malocclusion.

Psychosocial Impact of Malocclusion	Normative Treatment Need				
	IOTN-AC	IOTN-DHC	DAI	ICON	PAR
	10.9% (n=73)	47.8% (n=319)	30.6% (n=204)	37.9% (n=253)	11.4% (n=76)
Concerned					
No: 39.6% (*n* = 264);					
Yes: 60.4% (*n* = 403)					
Odds ratio	0.559	0.672	1.090	0.918	0.812
Standard error	0.284	0.169	0.184	0.171	0.259
*p*-value	0.040*∗*	0.019*∗*	0.638	0.618	0.421
Confidence interval	0.320-0.974	0.482-0.936	0.761-1.563	0.657-1.284	0.489-1.348

Smiling					
No: 66.0% (*n* = 440);					
Yes: 34.0% (*n* = 227)					
Odds ratio	0.546	0.937	1.335	1.074	0.688
Standard error	0.259	0.172	0.194	0.174	0.252
*p*-value	**0.020** **∗**	0.707	0.138	0.682	0.138
Confidence interval	0.329-0.907	0.669-1.314	0.912-1.954	0.763-1.512	0.420-1.127

Teased					
No: 52.2% (*n* = 348);					
Yes: 47.8% (*n* = 319)					
Odds ratio	0.647	1.071	0.762	0.855	1.228
Standard error	0.259	0.164	0.180	0.166	0.249
*p*-value	0.093	0.677	0.132	0.344	0.409
Confidence interval	0.389-1.076	0.776-1.476	0.535-1.085	0.617-1.183	0.754-2.001

*∗p* < 0.05.

**Table 4:** Associations between normative treatment need and sociodemographic factors.

Socio-demographic Factors	Normative Treatment Need				
	IOTN-AC	IOTN-DHC	DAI	ICON	PAR
	10.9% (n=73)	47.8% (n=319)	30.6% (n=204)	37.9% (n=253)	11.4% (n=76)
Gender					
Male: 50.8% (*n* = 339)					
Female: 49.2% (*n* = 328)					
*p*-value	0.356	**0.015** **∗**	**0.033** **∗**	0.194	0.054
Confidence interval	0.477-1.305	0.485-0.924	0.482-0.970	0.899-1.694	0.992-2.655

Education					
College: 21.0% (*n* = 140)					
Below college: 79.0% (*n* = 527)					
*p*-value	0.953	0.020*∗*	0.153	0.636	0.022*∗*
Confidence interval	0.572-1.694	0.461-0.935	0.509-1.112	0.651-1.300	0.303-0.911

Income					
>20000: 43.3% (*n* = 289) **≤**20000:					
56.7% (*n* = 378)					
*p*-value	0.131	0.198	0.828	0.739	0.662
Confidence interval	0.883-2.597	0.891-1.748	0.666-1.384	0.675-1.322	0.552-1.459

*∗p* < 0.05.

**Table 5:** Associations between the psychosocial impact of malocclusion and sociodemographic factors.

Psychosocial Impact of Malocclusion	Socio-demographic Factors		
	Gender	Education	Income
	Male: 50.8% (*n* = 339)	Below college: 79.0% (*n* = 527)	≤20,000 HKD: 56.7% (*n* = 378)
	Female: 49.2% (*n* = 328)	College: 21.0% (*n* = 140)	> 20,000 HKD: 43.3% (*n* = 289)
Concerned			
No: 39.6% (*n* = 264);			
Yes: 60.4% (*n* = 403)			
Odds ratio	1.063	1.246	0.996
Standard error	0.164	0.179	0.190
*p*-value	0.709	0.221	0.984
Confidence interval	0.771-1.465	0.876-1.771	0.686-1.447

Smiling			
No: 66.0% (*n* = 440);			
Yes: 34.0% (*n* = 227)			
Odds ratio	0.962	1.116	1.039
Standard error	0.168	0.182	0.197
*p*-value	0.817	0.547	0.845
Confidence interval	0.692-1.337	0.781-1.594	0.707-1.528

Teased			
No: 52.1% (*n* = 348);			
Yes: 47.9% (*n* = 319)			
Odds ratio	0.947	1.124	1.080
Standard error	0.160	0.174	0.186
*p*-value	0.734	0.501	0.680
Confidence interval	0.693-1.295	0.800-1.579	0.750-1.556

## Data Availability

The datasets used and/or analyzed during this study are available from the corresponding author on reasonable request.
